# Visualization of Abscess Formation in a Murine Thigh Infection Model of *Staphylococcus aureus* by ^19^F-Magnetic Resonance Imaging (MRI)

**DOI:** 10.1371/journal.pone.0018246

**Published:** 2011-03-24

**Authors:** Tobias Hertlein, Volker Sturm, Stefan Kircher, Thomas Basse-Lüsebrink, Daniel Haddad, Knut Ohlsen, Peter Jakob

**Affiliations:** 1 Institute for Molecular Infection Biology, University of Würzburg, Würzburg, Germany; 2 Department of Experimental Physics 5, University of Würzburg, Würzburg, Germany; 3 Institute of Pathology, Uniklinik Würzburg, Würzburg, Germany; 4 Magnetic Resonance Bavaria, Würzburg, Germany; Cornell University, United States of America

## Abstract

**Background:**

During the last years, ^19^F-MRI and perfluorocarbon nanoemulsion (PFC) emerged as a powerful contrast agent based MRI methodology to track cells and to visualize inflammation. We applied this new modality to visualize deep tissue abscesses during acute and chronic phase of inflammation caused by *Staphylococcus aureus* infection.

**Methodology and Principal Findings:**

In this study, a murine thigh infection model was used to induce abscess formation and PFC or CLIO (cross linked ironoxides) was administered during acute or chronic phase of inflammation. 24 h after inoculation, the contrast agent accumulation was imaged at the site of infection by MRI. Measurements revealed a strong accumulation of PFC at the abscess rim at acute and chronic phase of infection. The pattern was similar to CLIO accumulation at chronic phase and formed a hollow sphere around the edema area. Histology revealed strong influx of neutrophils at the site of infection and to a smaller extend macrophages during acute phase and strong influx of macrophages at chronic phase of inflammation.

**Conclusion and Significance:**

We introduce ^19^F-MRI in combination with PFC nanoemulsions as a new platform to visualize abscess formation in a murine thigh infection model of *S. aureus*. The possibility to track immune cells in vivo by this modality offers new opportunities to investigate host immune response, the efficacy of antibacterial therapies and the influence of virulence factors for pathogenesis.

## Introduction

In recent years, several in vivo imaging modalities were developed to monitor infections in real-time and non-invasively. Bioluminescent imaging (BLI) enables detection of luminescent bacteria in several animal models [1], [2]. Moreover, Fluorine-18-deoxyglucose and fluorinated pyrimidine nucleosides have been applied to visualize sites of inflammation or infection by positron emission tomography (PET) [3], [4]. Another imaging modality is magnetic resonance imaging (MRI), which has been adapted especially in combination with contrast agents to image edema and inflammation. In this context, ^1^H-based MRI has been proven to be suitable to detect edema pattern in muscoskeletal [5], [6] and inflammatory lesions in lung infection models [7], [8]. However, inflammatory reactions like accumulation of liquid in the interstitial space are unspecific and lead to inaccuracy in diagnosis of infection in postoperative and posttraumatic situations [9]. Nonetheless, MRI is a preferable modality because it is able to provide soft tissue contrast and higher spatial resolution compared to other imaging technologies such as BLI or PET. Therefore, the development of a MRI contrast agent to visualize inflammation and to monitor the efficacy of therapeutic measures would be of great benefit.

Recently, USPIO (ultrasmall paramagnetic ironoxides) nanoparticles were evaluated as inflammation specific contrast agents [5]. They are phagocytosed after intravenous administration by monocytes/macrophages in the bloodstream. The phagocytes migrate then to the infection site, in turn leading to accumulation of ironoxide nanoparticles. Since the susceptibility effects of iron particles results in signal reduction on MR images, a differentiation of inflammatory area, edema pattern and surrounding tissue becomes visible [5], [10]. Previous work with USPIOs during acute or chronic phase of *S. aureus* soft tissue infection revealed a signal reduction at the abscess wall in T_2_ or T_2_* weighted MRI [10]. However, since superparamagnetic agents lead to signal extinction in MR images, the identification of affected areas is sometimes challenging. Furthermore, USPIOs are not suitable to visualize tissue changes in organs like the lung, which appear dark on conventional MR images.

Recently, a new class of MRI contrast media appeared: perfluorocarbon nanoemulsions. They are chemically and biologically inert synthetic molecules [11]. Importantly, the fluorine-19 nucleus is very useful for MRI due to its favorable gyromagnetic ratio (signal sensitivity of 89% in comparison to ^1^H). Furthermore, the very low biological abundance of ^19^F recommends its use as a MRI marker, since administered perfluorocarbon contrast media deliver unambiguous signals in vivo [12]. In one approach, PFCs have been used to label cells ex vivo and track their migration after administration in vivo. The migration of T cells in a murine model of diabetes [13] or the migration of dendritic cells to lymph nodes [14] was visualized by this modality. Another approach was the administration of PFCs into the blood stream, where they are phagocytosed by the monocyte/macrophage system and to a lower extend by neutrophils [15], [16]. The subsequent accumulation of these phagocytic cells at the site of inflammation after cardiac, cerebral ischemia [15] or pneumonia [16] could be visualized by ^19^F-MRI. However, to the best knowledge of the authors, PFC nanoemulsions have not been used yet to target, visualize and monitor infections.

Consequently, the purpose of this study was to evaluate the capability of PFC nanoemulsions to visualize and monitor infections. Therefore, we applied a basic animal model of *Staphylococcus aureus* infection and imaged the accumulation of ^19^Fluorine at the site of infection following PFC administration during acute and chronic phase of infection.

## Results

### T_2_ weighted MRI visualized the edema pattern caused by infection

**Figure 1:**
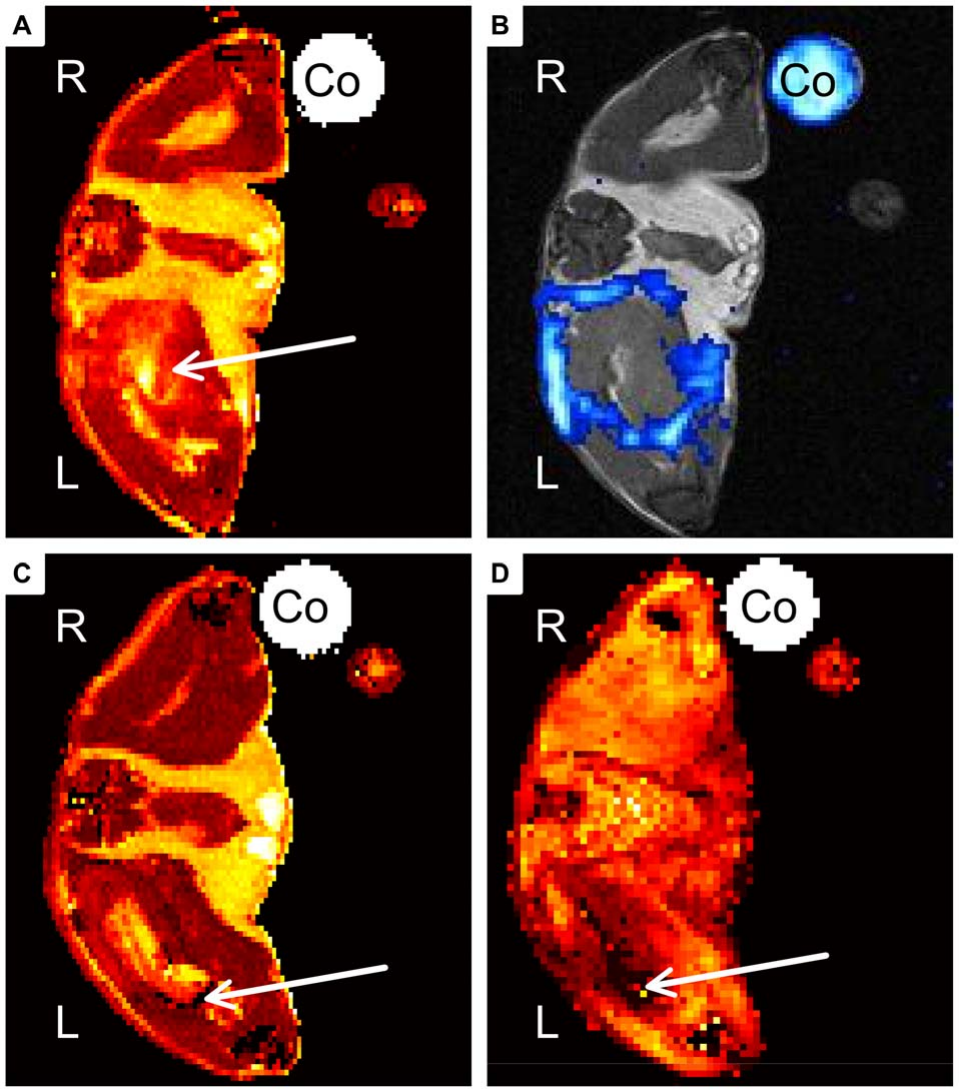
Representative MR Images of acute soft-tissue infection. A, B) Mouse received PFC at day 2 post infection. Images were recorded 24 h after administration. C, D) Mouse received CLIO at day 2 post infection and was imaged 24 h after administration. A) Transverse T_2_ map of PFC group mouse shows hyperintensity within infected muscle (arrow). B) ^19^F-SSFP CSI overlay on ^1^H-TSE image of PFC treated mouse shows strong accumulation of PFC at the rim of abscess area. C) Transverse T_2_ map of CLIO group mouse shows hyper-intense area diffusely circumscribed by dark spots (arrow). D) T_2_* weighted image shows diffusely distributed susceptibility effects in the infected muscle (arrow). (Co: Control tube filled with PFC dilution, R: right side, L: left side of the imaged mouse).

**Figure 2:**
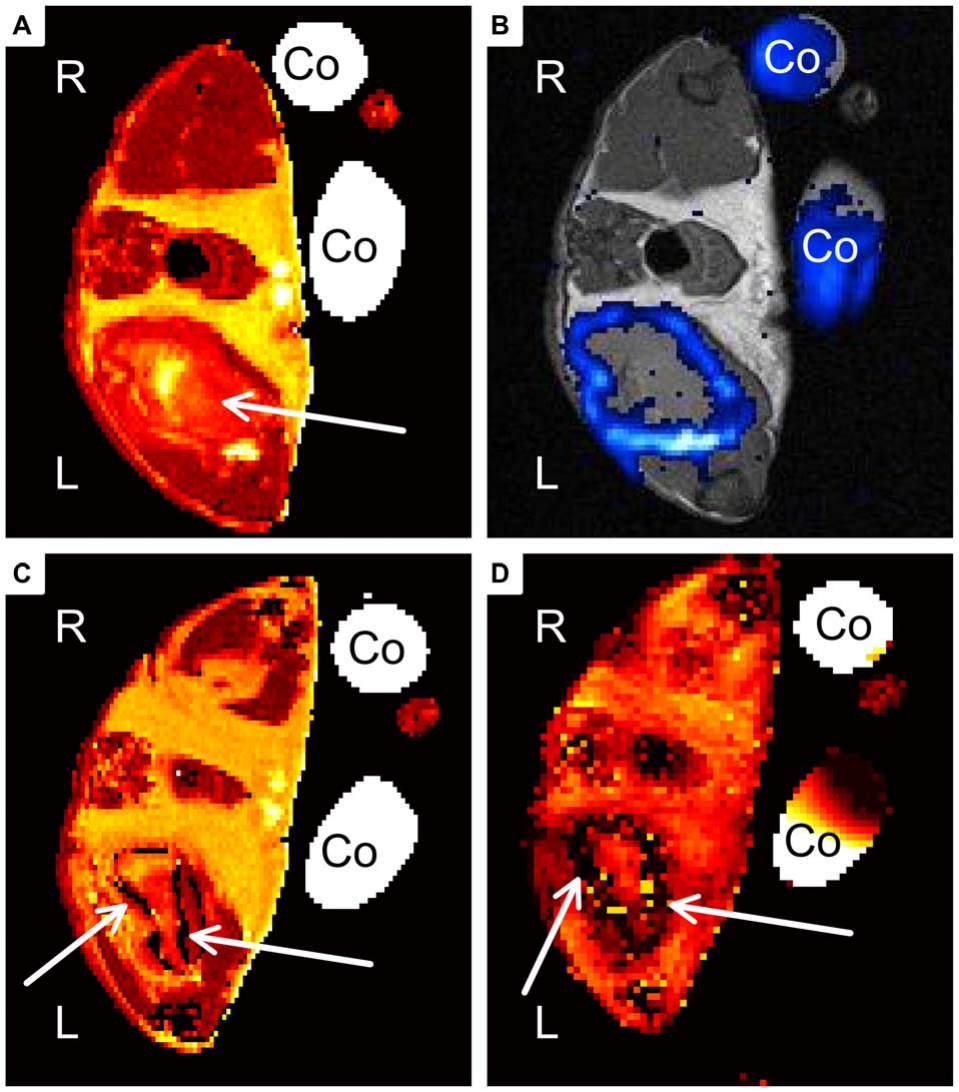
Representative MR Images of chronic soft-tissue infection. A, B) Mouse received PFC at day 8 post infection. Images were recorded 24 h after administration. C, D) Mouse received CLIO at day 8 post infection and was imaged 24 h after administration. A) Transverse T_2_ map of PFC group mouse shows large hyper-intensity area within infected muscle (arrow). B) ^19^F-SSFP CSI overlay on ^1^H-TSE image of PFC group mouse shows strong accumulation of PFC at the rim of abscess area. C) Transverse T_2_ map of CLIO group mouse shows hyper-intense area circumscribed by a dark rim (arrows). D) T_2_* weighted image shows strong susceptibility effects around the infected muscle in a hollow sphere pattern (arrows (Co: Control tube filled with PFC dilution, R: right side, L: left side of the imaged mouse).

We recorded T_2_ maps to visualize the edema pattern caused by *S. aureus* infection in the thigh muscle. It was shown previously that T_2_ values increase at the site of infection during inflammatory response [5], [6], [10]. All imaged mice developed increased T_2_ values in the infected left thigh muscle, which were visualized by T_2_ maps (Fig. 1A/C, Fig. 2A/C). The edema pattern appeared diffuse in all imaged mice and defined no clear border to surrounding muscle tissue. Injection of sterile 0,9% NaCl-solution into the right thigh muscle did not lead to increased signal intensity in T_2_ maps. Mice that received CLIO particles at acute phase of inflammation showed diffuse arranged ‘dark’ spots of signal decrease at the rim of the edema area (Fig. 1C). They are induced by signal distortion effects caused by the ironoxide nanoparticles. The administration of CLIO nanoparticles at chronic phase of inflammation lead to a ‘dark’ rim of signal distortion around the edema area (Fig. 2C).

### PFC accumulates in the shape of a hollow sphere around the edema area at acute phase

**Figure 3:**
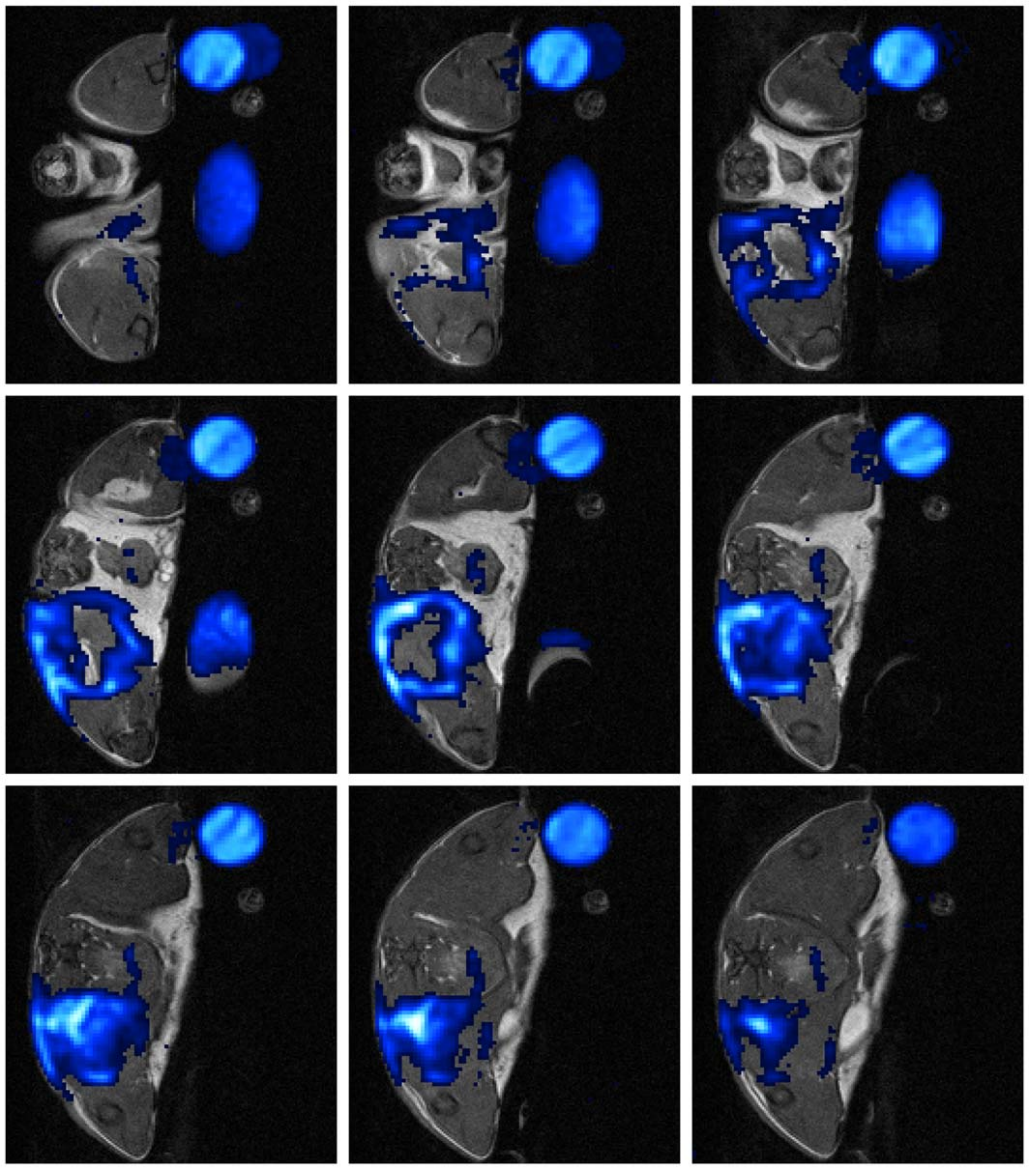
Representative MR-slice series showing the hollow sphere formed by PFC at acute phase. The depicted mouse received PFC at day 2 after start of infection. Images were recorded 24 h after administration. ^19^F-SSFP CSI overlay on ^1^H-TSE image of PFC group mouse shows strong accumulation of PFC at the rim of the abscess area.(Slice Thickness: 1 mm, FOV 25×25 mm^2^, Co: Control tube filled with PFC dilution, R: right side, L: left side of the imaged mouse).

To visualize the accumulation of PFC nanoparticles at the site of infection, ^19^F-SSFP CSI images were recorded and overlayed on ^1^H-TSE images which were used as anatomical reference. PFC nanoemulsion was administered intravenously at day 2 post infection and the mice were imaged 24 h later to analyze the accumulation of ^19^F at the site of infection during acute phase of inflammation. The PFC nanoparticles accumulated in all investigated mice in the shape of a hollow sphere at the rim of the edema pattern, which was defined by increased T_2_ values in comparison to surrounding muscle tissue (Fig. 3, Fig. 1B). No accumulation of PFC could be detected in the center of the abscess or in the right control thigh muscle, which was inoculated with sterile 0,9% NaCl-solution. Furthermore, no ^19^F signal was detected in infected mice without the administration of PFC nanoparticles (data not shown).

### CLIO accumulates diffuse around edema area at acute phase

We administered CLIO nanoparticles at day 2 post infection and imaged the mice 24 h later to compare the accumulation of PFC and ironoxide nanoparticles during acute phase of inflammation. The T_2_ maps, which were recorded to visualize the edema area, showed sparse and diffuse susceptibility effects at the rim of the edema pattern (Fig. 1C). T_2_* maps showed the signal distortion caused by accumulation of ironoxide nanoparticles at the edema rim (Fig. 1D). CLIO particles accumulated in all investigated mice only sparse and diffuse around the edema area and defined no clear border of the abscess area. No accumulation of CLIO could be visualized in the control thigh muscle.

### PFC and CLIO accumulate in the shape of a hollow sphere at chronic phase

**Figure 4:**
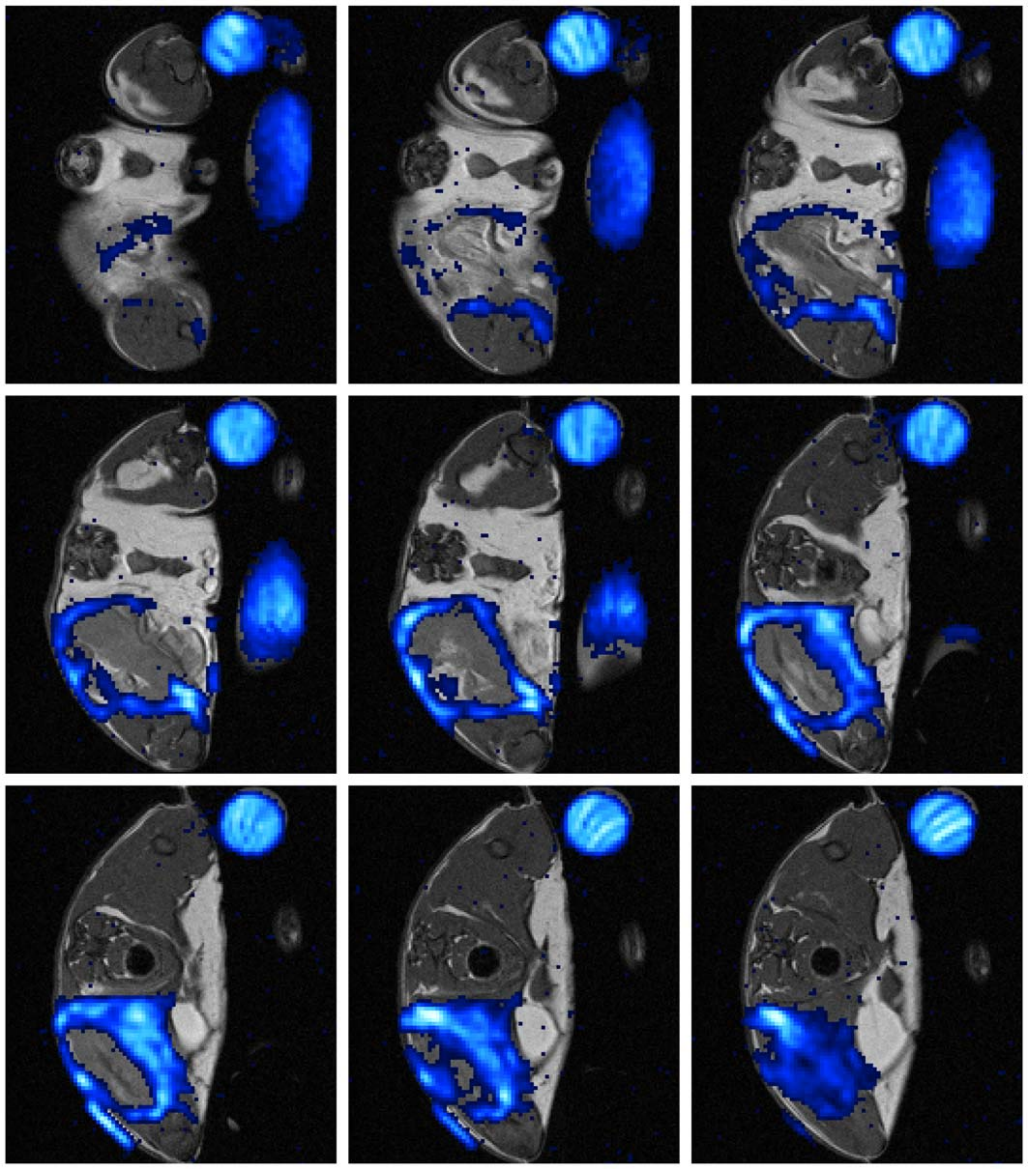
Representative MR-slice series showing the hollow sphere formed by PFC at chronic phase. The depicted mouse received PFC at day 8 post infection. Images were recorded 24 h after administration. ^19^F-SSFP CSI overlay on ^1^H-TSE image of PFC group mouse shows strong accumulation of PFC at the rim of abscess area. (Slice Thickness: 1 mm, FOV 25×25 mm^2^, Co: Control tube filled with PFC dilution, R: right side, L: left side of the imaged mouse).

To investigate and compare the accumulation of PFC and CLIO nanoparticles at the abscess area during chronic phase of inflammation, PFC or CLIO was inoculated intravenously at day 8 post infection and the mice were imaged 24 h later. T_2_ and T_2_* maps of all mice, that received CLIO, revealed strong signal extinction at the abscess rim of the infected muscle and thus accumulation of ironoxide nanoparticles (Fig. 2C/D). ^19^F signal of all PFC group mice showed strong accumulation at the rim of the edema area (Fig. 2B) similar to the accumulation pattern of ironoxide in CLIO group mice. CLIO as well as PFC (Fig. 4) accumulated in the shape of a hollow sphere at the border, but never in the center of the edema pattern. Neither PFC nor CLIO administration lead to a detectable signal in the control muscle. Thereby, differentiation of edema area and surrounding muscle tissue becomes possible by the use of CLIO as contrast agent as well as with PFC.

### Time course imaging of PFC accumulation

**Figure 5:**
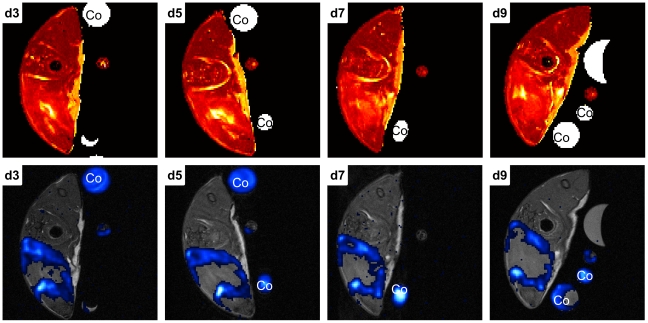
Representative MR-slice series showing the time course of PFC accumulation following administration at acute phase. Mouse received PFC at day 2 p.i. and was imaged every 48 h beginning at d3 p.i.. The upper row of images shows T_2_ weighted MR-slices, the lower row the corresponding ^19^F-SSFP CSI overlays on ^1^H-TSE images. ^19^F signal could be visualized in all measurements until d9 p.i. (end point).

We administrated PFC into a fifth group of infected animals 48h p.i. to visualize changes in the ^19^F accumulation pattern over time (Fig. 5). Imaging led to an equal pattern at d3 p.i. compared to acute group mice. Further measurements every 48 h demonstrated stability of ^19^F signal around the abscess area until the end point of this experiment (d9 p.i.).

### Histological examination of imaged muscles

**Figure 6:**
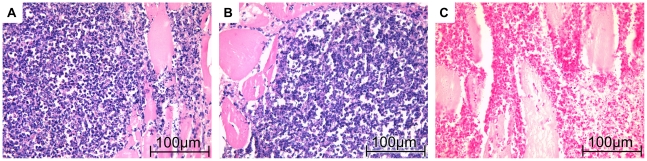
Histological appearance of acute soft-tissue infection. A, C) Mice received CLIO at day 2 post infection and were imaged 24 h after administration. The muscle was recovered immediately after imaging. B) Mice received PFC at day 2 post infection. Images were recorded 24 h after administration. The muscle was recovered immediately after imaging. A) HE-staining shows that infiltrate in CLIO group mice consists mainly of granulocytes. C) Iron-specific staining demonstrates iron deposits in granulocytes and macrophages (iron appears blue). B) HE-staining of PFC group mouse reveals strong immune cell infiltrate mainly consisting of granulocytes.

**Figure 7:**
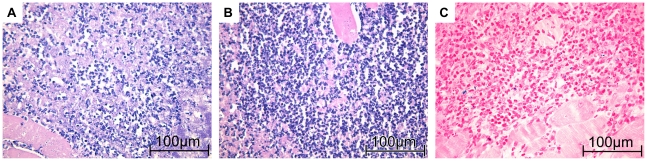
Histological appearance of chronic soft-tissue infection. A, C) Mice received CLIO at day 2 post infection. Images were recorded 24 h after administration. The muscle was recovered immediately after imaging. B) Mice received PFC at day 2 post infection and were imaged 24 h after administration. The muscle was recovered immediately after imaging. A) Macrophages and Granulocytes are embedded in granulation tissue at the abscess rim of CLIO group mice. C) Iron-specific staining demonstrates iron deposits in granulocytes and macrophages (iron appears blue). B) HE-staining of PFC group mice reveals macrophages and granulocytes in fibroblast-enriched granulation tissue similar to CLIO group mice.

HE staining of muscles from acute phase groups showed a very dense infiltrate of neutrophils at the abscess rim with some interspersed macrophages. They surrounded a central necrotic area. No difference of immune cell composition or density between PFC and CLIO group was observable (Fig. 6A/B). Chronic phase of inflammation was characterized by a bulky central necrosis encircled by a dense layer of granulocytes, macrophages and fibroblast-enriched granulation tissue. No difference in immune cell content or abscess size could be delineated between PFC and CLIO group mice (Fig. 7A/B).

Iron specific staining revealed cellular deposits of iron primarily in macrophages, but also in some neutrophils. Comparison of histological slices derived from acute (Fig. 6C) and chronic (Fig. 7C) inflammation groups stained with Berlin blue iron staining revealed stronger accumulation of iron-loaded macrophages during chronic phase of inflammation.

## Discussion

The aim of this study was to evaluate PFC nanoparticles as a new class of contrast media to target and visualize sites of bacterial infection. Therefore, we chose a murine thigh abscess model of *S. aureus* due to its localized and bulky abscess formation. It was shown before, that this animal model mirrors acute and chronic phase of inflammation during course of infection [10]. The acute phase of inflammation was thereby characterized by strong immigration of neutrophils to the infection site and influx of liquid and plasma components into the interstitial space [10]. Our results are consistent with previous studies which observed that the emerging edema pattern led to increased T_2_ values at the site of inflammation. However, one major limitation of edema visualization by T_2_ weighted MRI is that no sharp edges to localize exactly the area of infection can be defined. Therefore, a method to target pro-inflammatory immune cells, such as macrophages and neutrophils, is preferable to visualize the area and course of infection.

In accordance with this idea, USPIO particles were evaluated in recent years as inflammation specific contrast agents [5], [10], [17]. It was shown, that ironoxide nanoparticles accumulate at the abscess wall of *S. aureus* induced thigh muscle abscesses [5]. Histological staining of imaged muscles demonstrated intracellular deposits of ironoxide in macrophages. But even though USPIO particles accumulated very strongly during chronic phase of inflammation, and thereby lead to clear identification and localization of the abscess area, their application during acute phase resulted only in weak accumulation avoiding the determination of the abscess area [10]. Furthermore, the application of ironoxide nanoparticles is limited by the fact that they are usually detected by their local signal extinction. For these reasons, it is difficult to unequivocally determine accumulated ironoxide nanoparticles in tissues, which appear dark on MR images or in neighborhood of other signal distorting structures.

Perfluorocarbon (PFC) nanoparticles circumvent this problem by delivering unambiguous signals in vivo due to the low natural abundance of ^19^F [12]. As it has been demonstrated that they are phagocytosed by the monocyte/macrophage system and to a lower extend by granulocytes [15], [16], the idea of this study was to use PFCs instead of ironoxide nanoparticles to visualize infection sites by MRI.

We administered PFC nanoparticles during acute or chronic phase of inflammation and compared their accumulation pattern by MRI with that of ironoxide particles (CLIO). CLIO group mice showed accumulation of ironoxide in acute and chronic phase. The accumulation in acute phase was diffuse and failed to define the abscess area (Fig. 1D). In contrast, CLIO accumulated in chronic phase at the rim of the edema pattern, defined by higher T_2_ values, and formed a hollow sphere. PFC administration at acute phase (Fig. 1B) led to a partially open hollow sphere around the edema pattern and allowed the distinction of abscess area and surrounding muscle tissue. Furthermore, PFC accumulated in chronic phase of infection like CLIO characterized by forming a closed hollow sphere around the edema pattern. Clearly, PFC accumulation was easier to identify than ironoxide owing to their background-free visualization. However, we cannot exclude the possibility that PFC accumulated stronger than CLIO nanoparticles during acute phase. The time course evaluation of PFC accumulation after administration during acute phase illustrated the stability of ^19^F signal around the abscess area at least until d9 p.i.. Taken together, PFC is able to target and visualize abscess formation in a *S. aureus* thigh abscess model in both acute and chronic phase of infection, whereas CLIO can only visualize the abscess area in chronic phase but fails to define the abscess area in acute phase.

Histologic examination of imaged muscles revealed neutrophils and some macrophages in the area where CLIO and PFC accumulation has been detected by MRI in acute phase. Iron-specific staining shows a few intracellular iron deposits in macrophages and granulocytes. At chronic phase, iron-loaded macrophages, lymphocytes and some granulocytes could be detected. These data are in accordance to previous reports on accumulation of ironoxide around soft-tissue abscesses [5], [10]. Unfortunately, we could not visualize PFC accumulation by histology as to our best knowledge there is no method available to stain PFC nanoparticles in histological preparations. Importantly, previous studies on inflammation after cardiac and cerebral ischemia demonstrated uptake of PFCs by phagocytic immune cells [15], [16]. Furthermore, the accumulation pattern of PFCs in our infection model is congruent with CLIO accumulation around the abscess. Finally, the validity of our method was further confirmed by the fact that we could never visualize accumulation of ^19^F in control thigh muscles, which were treated with sodiumchlorid-solution. All these observations strongly suggest accumulation of ^19^F signal at the abscess wall owing to the uptake of PFCs by phagocytic immune cells and their subsequent migration to the site of infection.

In conclusion, we have demonstrated that PFC nanoemulsions accumulated in the shape of a hollow sphere at the abscess of a *S. aureus* murine thigh infection model in both acute and chronic phase of infection. The detected signal arises exclusively from the nanoparticles due to the low biological abundance of ^19^F and the localization of the signal could be correlated on an anatomical ^1^H-MR image, what in turn enables the distinction of abscess area and surrounding tissue. The new contrast media based MRI modality presented here can be used to visualize inflammation, but may be furthermore interesting to be applied to investigate host-pathogen interaction, the efficacy of anti-microbial therapy, and the course of infections in vivo non-invasively with high spatial resolution.

## Materials and Methods

### Ethic statement

All animal experiments were approved by the government of Lower Franconia (54-2531.01-42/06) and were performed according to the German animal protection law. All animals were kept in cages under standardized lighting conditions and had ad libitum access to food and water.

### Animal model

To evaluate the capability of PFC as contrast media to visualize sites of infection, we choose a murine thigh infection model of *S. aureus* because of the localized and bulky abscesses generated by this model [18]. 16 female Balb/C mice (18–22 g, Charles-River, Germany) were used for this study. While the animals received inhalation anesthesia (1,5% isoflurane in O_2_), 50 µl of bacterial suspension (1×10^8^ cfu per infection dose) in 0,9% NaCl-solution was inoculated into the left thigh muscle. To visualize unspecific contrast agent accumulation, 50 µl of sterile 0,9% NaCl-solution was injected into the right thigh muscle. Bacterial suspension was prepared by overnight incubation of *Staphylococcus aureus* Xen 29 (Xenogen, USA) in liquid B-medium. The bacteria were washed, resuspended in 0,9% NaCl-solution and diluted to a final concentration of 2×10^9^ colony-forming units per milliliter. The infection dose was measured by optical density and checked by plating dilutions on agar plates.

The mice were then assigned to one of five groups. Two groups of 5 animals each received perfluorocarbon nanoemulsion at acute or chronic phase of infection, whereas the two other groups of 3 animals each received CLIO nanoparticles. To evaluate accumulation of contrast agent during acute phase, PFC or CLIO was administered intravenously at day 2 post infection. The chronic phase accumulation was investigated by administration of PFC or CLIO at day 8 post infection. In each case, the mice were imaged 24 h after administration by MRI. The animals were sacrificed within 3 hours after imaging by administration of inhalable pure CO_2_.

To visualize the accumulation of PFC in time-course, we administrated PFC 48h p.i. and imaged the ^19^F content at the abscess area in a fifth group of mice (3 animals). The animals were imaged at several time points between d3 and d9 p.i..

### Histology

The infected thigh muscle was prepared anatomically, removed and fixed in 10% neutral buffered formalin solution (Sigma-Aldrich, Germany). The pathologic specimen were then embedded in paraffin blocks according to standard procedures and cut to 2 µm thick transversal slices. Sections were stained with haematoxylin-eosin to characterize soft-tissue infection and with Berlin blue staining to visualize iron content. The histologic findings were evaluated by a pathologist (S. K.) and compared to MR images.

### Contrast agents

The home-built dextran coated CLIO particles had a diameter of 30 nm while the core diameter was around 2–3 nm (Department of Inorganic Chemistry, University of Würzburg). It was emulgated in phosphate buffered saline (6.8%_o_ m/m) for injection. 16,8 mg Fe/kg bodyweight were injected intravenously into the CLIO group mice.

The ^19^F nanoemulsion core compound was perflourocarbon with a mean droplet diameter of approximately 145 nm. The 20% v/v emulsion (VS1000H, Celsense, Inc., Pittsburgh, PA, USA) was for direct intravenous injection. 100 µl of this emulsion was administered intravenously into PFC group mice.

### MRI measurements

Measurements were performed on a 7 Tesla Bruker Biospec System (Bruker BioSpin GmbH, Reinstetten, Germany) at room temperature with an adjustable double resonant (^1^H and ^19^F) home-built birdcage. The animals received inhalation anesthesia (1% isoflurane) during measurement and were placed in a home-built measurement container due to safety regulations.

^1^H reference images were done by a turbo spin echo (TSE) sequence (TE_eff_/TR: 13.4 ms/2500 ms; inter-echo time: 6.7 ms; turbo factor: 4; FOV: 25×25 mm^2^; matrix: 200×200; slice thickness (SI): 1 mm; 16 slices, NA: 2).

For T_2_-mapping images were acquired by a multi slice multi spin echo (MSME) sequence (TE/TR: 6 ms/6000 ms; NE: 40; FOV: 25×25 mm; matrix: 128×128; slice thickness (SI): 1 mm; 10 slices interlaced, NA: 1). For T_2_* mapping 3D multi gradient echo (MGE) scans (TE/TR: 2 ms/100 ms; inter echo time: 2.7 ms; NE: 16; FOV: 40×25×25 mm^3^; matrix: 128×80×80; NA: 2) were performed.

Afterwards the signal decay parameters were fitted pixel by pixel using a T_2_/T_2_* maximum likelihood estimation based on the Rice distribution [19].

Regarding ^19^F-MRI acquisition-weighted [20] 3D steady-state free precession CSI (SSFP-CSI) sequences [21] were performed with the same geometry as the ^1^H-TSE scans (Pulse shape: hermite; Pulse bandwidth: 5400 Hz; T_ACQ_/TR: 10.24 ms/13.6 ms; FOV: 25×25×16 mm^3^; spectral points: 512; acquired resolution/time equivalent: - matrix: 48×48×8; - NA: 4). For overlay-images the signal of the ^19^F peak was integrated and afterwards the dataset was zero-filled to a matrix of 200×200×16 pixels. Furthermore a threshold was applied to the ^19^F data and afterwards underlayed with the ^1^H-TSE data. For each measurement the threshold of the overlay image was adjusted individually. The overall protocol time was less than three hours.
